# Successful Surgical Management of an Advanced Abdominal Pregnancy in a Resource‐Limited Setting

**DOI:** 10.1155/cris/1054912

**Published:** 2026-09-23

**Authors:** Pietro Pasquini, Faraja Bernard Mwidete, Agnes Mlawa, Giacomo Antonini

**Affiliations:** ^1^ Division of Obstetrics and Gynecology, IRCCS Azienda Ospedaliero-Universitaria di Bologna, Bologna, Italy; ^2^ Department of Medical and Surgical Sciences (DIMEC), University of Bologna, Bologna, Italy, unibo.it; ^3^ Department of Gynecology and Obstetrics, Consolata Hospital Ikonda, Makete, Tanzania; ^4^ Chief Medical Officer at Consolata Hospital Ikonda, Makete, Tanzania; ^5^ Department of Surgery, Consolata Hospital Ikonda, Makete, Tanzania; ^6^ Department of Surgery, Ospedale di Perugia, Perugia, Italy, ospedale.perugia.it

**Keywords:** abdominal pregnancy, bowel resection, ectopic pregnancy, extrauterine pregnancy, limited resource setting

## Abstract

**Introduction:**

Abdominal pregnancy (AP) is a rare ectopic pregnancy (EP), often misdiagnosed, with no standardized treatment due to limited literature. Most APs result in early pregnancy loss, making advanced AP cases even rarer. We present a case of advanced AP in a limited resource setting.

**Case Presentation:**

A 23‐year‐old woman with a previous cesarean delivery presented with a 2‐month history of amenorrhea, chest pain, and lower abdominal pain. Despite a positive pregnancy test, pelvic ultrasound showed no intrauterine or EP but revealed a paracecal mass, raising suspicion of an appendiceal abscess. Because of financial constraints, no further diagnostic investigations were performed, and urgent exploratory laparotomy was undertaken. Surgery revealed abortive material, including fetal bone fragments consistent with a 21–23‐week pregnancy. Ischemic placental tissue infiltrated the serosa and deeper layers of the cecum and distal ileum, with active bleeding, deperitonealization, and partial bowel obstruction. An ileocecal resection with ileocolic anastomosis was therefore performed. Recovery was uneventful, with no complications at 30‐day and 18‐month follow‐up.

**Conclusion:**

High clinical suspicion for AP is crucial, especially in cases with inconclusive imaging. A tailored surgical approach to manage placental invasion can lead to positive outcomes even in complex cases of advanced AP.

## 1. Introduction

Abdominal pregnancy (AP), first defined by Studdiford in 1942, refers to implantation within the peritoneal cavity, excluding tubal, ovarian, or intraligamentous sites [1]. It may be classified as primary (direct peritoneal implantation) or secondary (migration from the fallopian tube) and as advanced or nonadvanced based on whether the gestational age exceeds 20 weeks. Potential implantation sites include the omentum, liver, spleen, Pouch of Douglas, major blood vessels, intestines, and pelvic side wall. Epidemiologically, AP is rare, accounting for ~1% of ectopic pregnancies (EPs), which themselves occur in ~2% of all pregnancies, with its true incidence likely underestimated. Risk factors overlap with other EPs and include prior pelvic surgery, infections, infertility, and a lower socioeconomic status. Diagnostically, ultrasound criteria by Allibone et al. include extrauterine location of the fetus and placenta, absence of uterine wall between the fetus and bladder, unusual fetal positioning, and minimal amniotic fluid [2]. There are no standardized guidelines for managing advanced AP, and structured protocols could improve maternal and neonatal outcomes.

This case was managed at Consolata Hospital Ikonda, a mission hospital in the Njombe region, Tanzania, operating in a limited‐resource setting.

## 2. Case Report

A 23‐year‐old woman presented with a 2‐month history of amenorrhea, chest pain, and lower abdominal pain. Her obstetric history was notable for one prior cesarean delivery. She had no significant medical history and no assisted reproductive technology (ART) or tubal surgery, and denied vaginal bleeding.

Physical examination showed a tender, mobile mass in the right iliac fossa; thoracic findings were unremarkable. Given the thoracic symptoms, a chest X‐ray and ECG were performed, both yielding negative results. Despite a positive pregnancy test, a tender right iliac fossa mass with mild leukocytosis and a paracecal mass on pelvic US raised suspicion of an appendiceal abscess.

An appendiceal abscess was suspected, and the patient was admitted. Blood tests showed mild anemia (Hb 8.4 g/dL), mild leukocytosis (WBC 11.4 × 10^9^/L), and thrombocytosis (PTL 559 × 10^9^/L). Given the patient’s preference due to financial limitations, no additional radiological or laboratory investigations were performed, including serum β‐HCG. Only routine preoperative blood tests were conducted. Consequently, the patient was scheduled for an urgent exploratory laparotomy.

**Figure 1:**
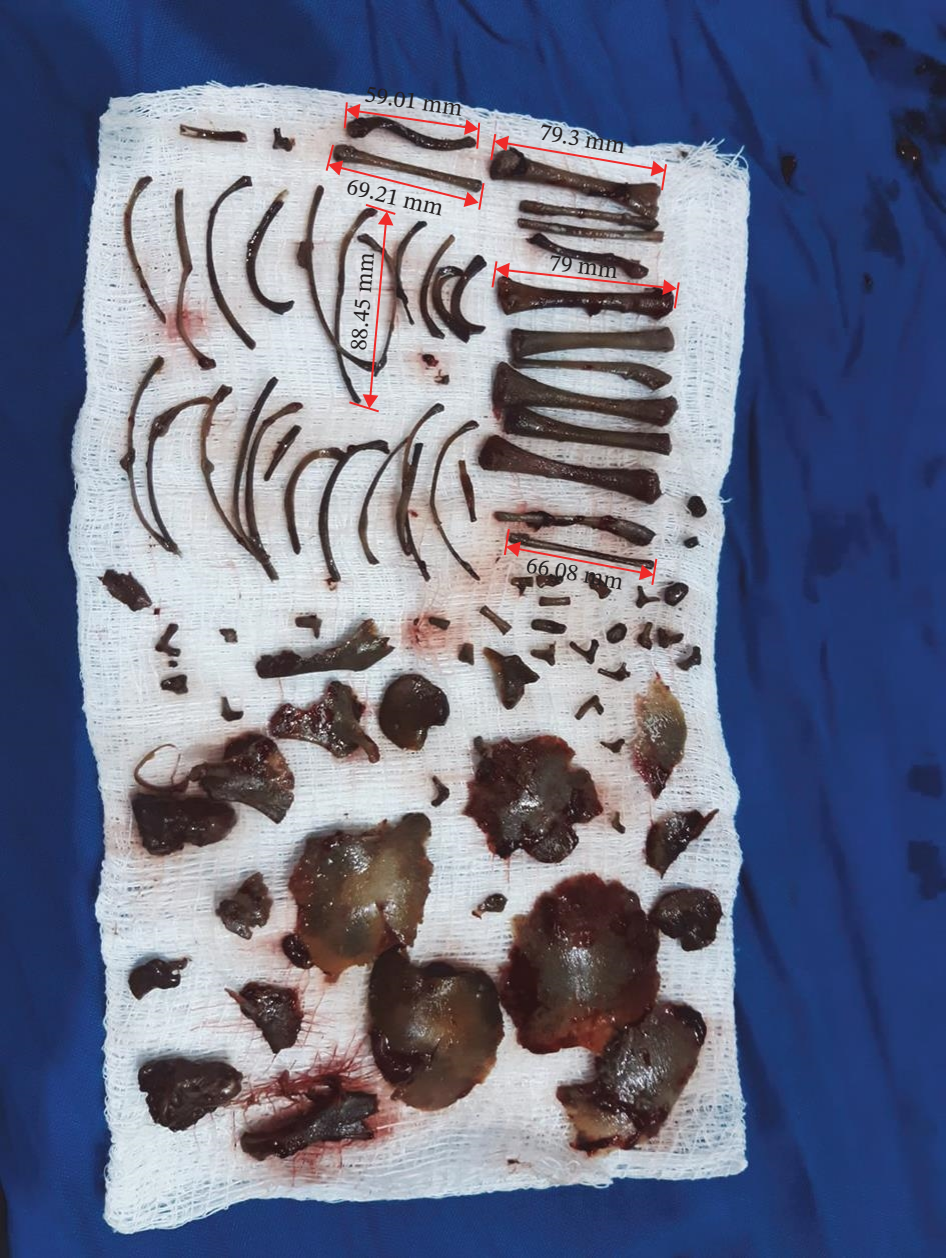
Multiple fetal bony fragments extracted from the abdominal cavity, displayed on a surgical swab. The upper left shows rib fragments; the upper right shows long bones (femur, humerus, tibia/fibula); the central area contains vertebral fragments and small bones; the lower portion shows flat bone fragments including cranial bones and a structure consistent with a scapula with a rudimentary acromial process. All fragments appear dark and necrotic, with no identifiable soft tissue. Red lines indicate measurements used to estimate gestational age of 21–23 weeks.

The day following the admission, an extended midline laparotomy was performed by the general surgery team. During surgery, 300 mL of serosanguinous fluid was aspirated, and dense adhesions were found involving the right anterior abdominal wall, cecum, omentum, distal ileal loops, the ipsilateral Fallopian tube and ovary, as well as the posterior uterine wall. Following meticulous dissection and adhesiolysis, multiple bony fragments were discovered and extracted from the right iliac fossa, Supporting Information (video). These were identified as abortive material corresponding to a fetus estimated to be between 21 and 23 weeks of gestation (Figure 1).

Ischemic placental remnants were observed infiltrating the serosa and deeper layers of the cecum and distal ileal loops with signs of ileal stasis. Although the mesenteric aspect of the loops appeared viable, deperitonealization following adhesiolysis, placental invasion, and partial luminal obstruction led to the decision to perform bowel resection. A resection of ~10 cm of the ileum and the entire cecum was performed, followed by an end‐to‐side ileocolic anastomosis. Reinforcing sutures were placed on the deperitonealized ileal segments. Two drains were placed: one in the pouch of Douglas and one in the right perianastomotic space. Although the estimated intraoperative blood loss was limited, one unit of packed red blood cells was transfused because the patient had pre‐existing anemia (Hb 8.4 g/dL) and was undergoing major abdominal surgery. The procedure lasted 1 h and 45 min.

Postoperatively, the patient was admitted to the ICU, where she received intravenous fluid resuscitation, antibiotic coverage with ceftriaxone and metronidazole for 5 days, and analgesia with paracetamol. Laboratory tests showed mild anemia (Hb 8.7 g/dL), leukocytosis (WBC 17.1 × 10^9^/L), thrombocytosis (PTL 465 × 10^9^/L), and hypokalemia (K^+^ 2.9 mmol/L). She was transferred to the ward on day 3 and discharged uneventfully on day 7. The 30‐day postoperative follow‐up was unremarkable: the wound had healed well, with no abdominal symptoms, bowel irregularities, or signs of surgical complications. A follow‐up pelvic ultrasound also yielded normal findings, with no evidence of intraperitoneal fluid collections or other abnormal findings.

The patient was contacted at 18 months postdischarge and confirmed being in good health. Despite having regular menstrual cycles and actively attempting to conceive, she has not conceived to date.

## 3. Discussion

We report a case of advanced AP managed surgically in a resource‐limited setting, a condition of exceptional rarity for which standardized management guidelines are still lacking due to the scarcity of reported cases, particularly in low‐resource environments. The diagnosis was confirmed retrospectively according to Studdiford criteria [1], which include the absence of uteroperitoneal fistula, entirely intraperitoneal pregnancy findings, bilaterally normal adnexa, and no signs of secondary implantation following initial primary tubal nidation.

The estimation of a gestational age of 21–23 weeks was based on the measurements of femur and skull bones [3–5], which, according to established definitions, qualifies this case as an advanced AP [6]. The findings support a diagnosis of aborted pregnancy: despite the intrinsic limitations of imaging, ultrasound showed no signs of an active pregnancy (no placenta, fluid, heartbeat, or fetal movements). Intraoperative exploration revealed only bony fragments, with no identifiable soft tissue, and mature placental tissue was absent, with only ischemic remnants at the level of the cecum and distal ileal loops.

The intraoperative findings do not align with the reported amenorrhea (a second‐trimester pregnancy compared to 2 months of amenorrhea). Therefore, it is reasonable to assume that the reported LMP was likely vaginal bleeding due to an EP, a typical feature of the condition [7].

Clinical and intraoperative findings (prior surgery, early coitarche, unprotected intercourse, adhesions, and bilateral tubal obstruction) suggest asymptomatic PID. As extensively documented in the literature, PID is a well‐known risk factor for infertility and EP [8]. Furthermore, its incidence is notably higher in nonindustrialized countries compared to industrialized ones [9]. To date, the patient reports having regular menstrual cycles and, despite active attempts to conceive, has not achieved pregnancy 18 months after surgery.

Although APs are often misdiagnosed in up to 50%–90% of cases according to the literature [10, 11], certain limitations in the preoperative management of this patient should be highlighted. The rapid urine pregnancy test (UPT) indicated the need for a pelvic ultrasound, which yielded negative results. The UPT outcome was initially interpreted as a false positive due to the test’s low diagnostic accuracy [12]. Given the amenorrhea and the misleading UPT result, a serum β‐HCG could have aided the diagnosis. Similarly, advanced imaging modalities such as CT or MRI, which are particularly effective in identifying extrauterine pregnancies, should also be considered essential. In fact, MRI can be used to diagnose an AP, and more importantly, MRI can help locate and identify the relationship between the placenta and the adjacent organs and tissues [13]. The limitations of the treatment can primarily be attributed to the limited‐resource healthcare setting and the counseling process. The delayed diagnosis was primarily attributed to the limited sensitivity and specificity of US imaging. However, due to the symptoms and the patient’s clear preference to avoid additional diagnostic evaluations, further clinical and imaging assessments were deferred, resulting in the decision of immediate surgical intervention.

Numerous case reports have documented situations where the placenta was left in situ without resulting in pathological sequelae [14]. Conversely, other cases reported complications such as infections [15], pre‐eclampsia‐related conditions [16], ascites [17], or intra‐abdominal postpartum bleeding [18], which necessitated a second surgical intervention [19]. Placental removal is debated, with no conclusive evidence supporting its necessity [14]. The choice, therefore, involves either removing the placenta, a high‐risk procedure requiring complex hemostasis and repair of the affected organ, or leaving it in situ, closely monitoring the patient in the postoperative period, and considering a second intervention if necessary [15]. As mentioned, the placenta was not clearly identified, although ischemic remnants and the appearance of the cecum and distal ileum suggested implantation at that level. Given the active bleeding and the evident compromised state of these organs, including intestinal stenosis and visible stasis of material in the distal ileum, a decision was made to excise the residual placental tissue through ileocecal resection, followed by an end‐to‐side anastomosis. Consistent with the findings of Machado et al., who reported a surgical management rate of 93.9% and a transfusion rate of 23.6% across 314 cases, our patient required surgical intervention with intraoperative transfusion [11]. Although the estimated intraoperative blood loss was contained, transfusion of a single unit was performed because the patient had pre‐existing anemia (Hb 8.4 g/dL) and was undergoing major abdominal surgery. Notably, despite the resource‐limited setting, the outcome in our case was favorable, with no postoperative complications, contrasting with an overall maternal mortality of 4.8% reported in the same review.

## 4. Conclusion

In female patients of reproductive age presenting with amenorrhea, abdominal pain, or vaginal bleeding, pregnancy should always be considered, with appropriate clinical, laboratory, and imaging investigations to exclude obstetric‐related conditions. In suspected AP, serum β‐HCG should be used to confirm and quantify the pregnancy, and ultrasonography should be complemented with CT or MRI to assess the relationships between the conceptus, placenta, and abdominal organs. Surgical intervention must be carefully planned to optimize outcomes and reduce complications, involving general surgery if abdominal organs are affected and preparing for significant blood loss with blood products. This case demonstrates that, even in resource‐limited settings where advanced diagnostics are unavailable, a high index of clinical suspicion and a prompt, tailored surgical approach can lead to favorable outcomes in this rare and life‐threatening condition.

## Author Contributions

All authors contributed to the study and manuscript preparation according to their respective roles. Pietro Pasquini was the principal author, responsible for patient follow‐up, study design, data collection, analysis, manuscript writing and revision, and final approval. Giacomo Antonini performed the surgical management and contributed to data collection, analysis, manuscript editing, and final approval. Agnes Mlawa contributed to surgical management, patient care, and data collection. Faraja Bernard Mwidete was responsible for postoperative management and follow‐up, data collection, manuscript drafting, and critical revision.

## Funding

The authors declare that no funds, grants, or other support were received during the preparation of this manuscript. Open access publishing was facilitated by Università degli Studi di Bologna, as part of the Wiley – CRUI‐CARE agreement.

## Consent

The patient provided written informed consent to participate in this study, with the consent process facilitated by a local physician fluent in the patient’s native language. The patient received a translation of the manuscript in her native language during the 18‐month follow‐up consultation and subsequently provided written informed consent for publication.

## Conflicts of Interest

The authors declare no conflicts of interest.

## Supporting Information

Additional supporting information can be found online in the Supporting Information section.

## Supporting information


**Supporting Information** Surgical removal of fetal bone fragments in an advanced abdominal pregnancy. The video highlights the extensive placental adhesion to the cecum and distal ileum, with dense adhesions and associated fecal stasis.

## Data Availability

The data that support the findings of this study are available from the corresponding author upon reasonable request.
